# Increase in retinal ganglion cells’ susceptibility to elevated intraocular pressure and impairment of their endogenous neuroprotective mechanism by age

**Published:** 2013-09-26

**Authors:** Hani Levkovitch-Verbin, Shelly Vander, Daria Makarovsky, Fabio Lavinsky

**Affiliations:** Sam Rothberg Ophthalmic Molecular Biology Laboratory, Goldschleger Eye Institute, Sheba Medical Center, Tel-Hashomer, Sackler Faculty of Medicine, Tel-Aviv University, Israel

## Abstract

**Purpose:**

To investigate age-associated changes in retinal ganglion cell (RGC) response to elevated intraocular pressure (IOP), and to explore the mechanism underlying these changes. Specifically, the effect of aging on inhibitor of apoptosis (IAP) gene family expression was investigated in glaucomatous eyes.

**Methods:**

IOP was induced unilaterally in 82 Wistar rats using the translimbal photocoagulation laser model. IOP was measured using a TonoLab tonometer. RGC survival was evaluated in 3-, 6-, 13-, and 18-month-old animals. Changes in the RNA profiles of young (3-month-old) and old glaucomatous retinas were examined by PCR array for apoptosis; changes in selected genes were validated by real-time PCR; and changes in selected proteins were localized by immunohistochemistry.

**Results:**

There were no significant IOP differences between the age groups. However, there was a natural significant loss of RGCs with aging and this was more prevalent in glaucomatous eyes. The number of RGCs in glaucomatous eyes decreased from 669±123 RGC/mm^2^ at 3 months to 486±114 RGC/mm^2^ at 6 months and 189±46.5 RGC/mm^2^ at 18 months (n=4–8, p=0.048, analysis of variance). The PCR array revealed different changes in proapoptotic and prosurvival genes between young and old eyes. The two important prosurvival genes, IAP-1 and X-linked IAP (XIAP), acted in opposite directions in 3-month-old and 15-month-old rats, and were significantly decreased in aged glaucomatous retinas, while their expression increased significantly in young glaucomatous eyes. P53 levels did not vary between young glaucomatous and normal fellow eyes, but were reduced with age. B-cell leukemia/lymphoma 2 (Bcl-2) family members and tumor necrosis factor (TNF)-α expression were unaffected by age. Immunohistochemistry results suggested that the sources of changes in IAP-1 protein expression are RGCs and glial cells, and that most XIAP secretion comes from RGCs.

**Conclusions:**

Decreased IAP-1 and XIAP gene expression in aged eyes may predispose RGCs to increased vulnerability to glaucomatous damage. These findings suggest that aging impairs the endogenous neuroprotective mechanism of RGCs evoked by elevated IOP.

## Introduction

Aging is a multifaceted process associated with several functional and structural deficits in the retina, including changes in blood flow [1], mechanical damage and axonal flow [2,3], mitochondrial dysfunction [4,5], and increased reactive oxygen species and oxidative stress, which may lead to genomic instability and DNA mutations with reduced survival [6-11].

Improvements in health care have increased human life expectancy, and it is estimated that about 80 million people will have glaucoma worldwide by 2020 [12]. Our understanding of how old age predisposes people to glaucoma is poor. It affects 1 in 200 individuals up to 50 years of age, and 1 in 10 individuals over 80 years of age. This age-associated increase in glaucoma prevalence is not accompanied by a corresponding increase in intraocular pressure (IOP) [13]. A few studies have suggested that age-related changes might play a role in glaucomatous optic neuropathy such that the retina itself and/or the optic nerve has an altered susceptibility to elevated IOP or other stress injuries [14,15].

It was recently shown that susceptibility to axonal transport deficits increases with age in DBA/2 mice and that this change is not necessarily associated with elevated IOP [16,17]. It was also found that severity of injury from the same ischemic insult was greater in optic nerves of older mice (≥12 months) compared to young optic nerves [18].

Aging is recognized as being a major risk factor for the development and progression of glaucoma, but the mechanism underlining this finding remains unclear [19-21]. In this study, we investigated whether increased age predisposes retinal ganglion cells (RGCs) to increased glaucomatous damage. In addition, we explored potential predisposing factors in search of novel protective and therapeutic measures against these processes.

## Material and Methods

### Experimental glaucoma

Wistar rats (3 to 18 months old) were used in accordance with the Association for Research in Vision and Ophthalmology Statement for Use of Animals in Ophthalmic and Vision Research in protocols approved and monitored by the Animal Care Committee of the Tel-Aviv University School of Medicine. Elevated IOP was induced in one eye of 82 animals using the translimbal photocoagulation laser model [22]. IOP measurements were taken immediately before and 1 day after each treatment, and then weekly with a TonoLab tonometer (TioLat, Helsinki, Finland).

### Labeling and counting of retinal ganglion cells

Retrograde labeling of RGCs with fluorogold (Fluorochrome, Inc., Englewood, CO) was performed bilaterally into the superior colliculi, as described previously [23]. Briefly, the rats were anesthetized, the scalp was exposed and holes approximately 2 mm in diameter were drilled in the skull 4 mm posterior to bregma, with a dentist’s drill (Dremel, Racine, WI) on both sides of the midline raphe. A stereotactical injection of FG was directly applied to each superior colliculus. The overlying skin was sutured and antibiotic ointment applied externally. The eyes were enucleated 10 days after labeling and 10 weeks after the first laser treatment, and flat retinal mounts were prepared.

Labeled RGCs were viewed with a fluorescence microscope and counted with a 40 super wide field objective along eight radii in four directions centered on the position of the optic nerve head. Four fields were counted along each radius, yielding a total of 32 fields per retina. The counting process was carried out by a masked experienced observer. We refer here to the RGCs as those identified by their FluoroGold (FG) staining and by their size and morphology. It is possible that some of the cells identified as RGCs were non-neuronal cells, although we believe this rare occurred, given that blood vessel cells and astrocytes have very distinctive morphologies.

The area of each field in our microscope is 0.34 mm^2^, yielding a total counted area of 10.88 mm^2^, which is a 21% sample of the average 50.1 mm^2^ Wistar rat retina. The total number of surviving RGCs per retina was calculated by multiplying the mean density of RGCs by the total retinal area. The number of RGCs in each retina was compared to a control retina to yield the survival rate. Data are presented as means ± standard error of the mean.

### Quantitative polymerase chain reaction array for apoptosis

RT [2] Profiler^TM^ PCR Arrays (Catalog # PARN-012 SABiosciences, Frederick, MD) was performed to check for expression of genes involved in facets of apoptosis. The array was done in glaucomatous eyes and control fellow eyes of young and old rats (3–18 months old; n=12 rats, pull of 4 animals for each PCR array). Each 96-well RT2 Profiler^TM^ PCR Array contains 84 wells for different genes related to apoptosis cascade, five wells with assays for different housekeeping genes, a genomic DNA (gDNA) control, three replicate reverse transcription controls, and three replicate positive PCR controls. Data were analyzed with the web-based PCR Array.

Total RNA was extracted from retinas dissected after 8 days using the Qiagen RNeasy mini kit (Qiagen, Valencia, CA). RNA quantity and purity was determined using the Nanodrop ND-2000 (Nanodrop Technologies, Wilmington, DE). RNA was reverse transcribed using the RT2 First Strand Kit (SABiosciences), Real-time quantitative PCR (qPCR) was performed using the RT [2] SYBR Green qPCR Master Mix (SABiosciences). Next, samples were aliquoted on the rat apoptosis PCR array. All steps were carried out according to the manufacturer’s protocol for the ABI Prism 7000 Sequence Detection System.

### Real-time reverse transcription polymerase chain reaction

Message levels of selected genes were examined by qPCR to verify array results. Several genes that were not on the microarray but were of particular interest to us were also examined. Total RNA was extracted from retinal samples of 3- and 15-month-old rats using TRIZOL (Invitrogen, Frederick, MD). One microgram of extracted RNA was reverse transcribed using an RT kit (Thermo Scientific, Epsom Surrey, UK), and real-time PCR was performed using the Platinum® SYBR® Green Two-Step qRT-PCR Kit with the ROX system (Invitrogen) in the ABI/Prism 7900HT Sequence Detector System (Applied Biosystem, Invitrogen). β-Actin messenger RNA (mRNA) was used as an endogenous control. Primers were purchased from Sigma (Sigma-Aldrich, Rehovot, Israel; Table 1.)

**Table 1:** Primers used for qPCR analysis of gene expression

**Primer (5’-3’)**	**Gene**
F: ATAACCGGGAGATCGTGAG R: CAGGCTGGAAGGAGAAAGATG	Bcl-2 GeneID:24224
F: TGTGCATCTGGGCCCTG R: CTGACCGTCCTGTAGTTCTCA	IAP-1 GeneID:78971
F: GTTCCGAGAGCTGAATGAGG R: TTTTATGGCGGGACGTAGAC	p53 GeneID:24842
F: GGTGAGTCGGATTGCAAG R: GGCAGTTAGGGATCTCCA	Bcl-xl GeneID:24888
F: CTCCCAGAAAAGCAAGCA R: CCTCTGCCAGTTCCACAAC	TNF-α GeneID:24835
F: GACAAATGTCCCAT R: CTAATGGACTGCGA	XIAP GeneID:63897
F: GCTACAGCTTCTCCACCACA R: TCTCCAGGGAGGAAGAGGAT	β-Actin GeneID:81822

### Immunohistochemistry

The eyes of each animal were enucleated and cryopreserved in sucrose/ optimal cutting temperature (OCT) compound (Sakura Finetek, USA Inc., Torrance, CA). Ten micrometer thick cryosections were collected onto Superfrost Plus slides. At least three sections from each eye were examined. For IAP, X-linked IAP (XIAP), Thy 1, a marker of RGC, and glial fibrillary acidic protein (GFAP), sections were incubated with goat antirat IAP (1:100, Santa Cruz Biotechnology), goat anti-XIAP (1:100, R&D Systems, Minneapolis, MN), mouse antirat Thy 1 (1:100, Biolegend, San Diego, CA), and mouse anti-GFAP (1:500, mouse monoclonal: Sigma Aldrich; rabbit polyclonal: Millipore, Billerica, MA). The secondary antibody was Alexa Fluor 633 or 488 conjugated antigoat IgG 1:500, Alexa Fluor 568 antirabbit 1:500, or Alexa Fluor 488 or 633 antimouse 1:500 (Invitrogen). Negative controls included nonimmune serum of the same species as the primary antibody at the same protein concentration with secondary antibody only.

Confocal images were acquired with a Zeiss LSM 750 (Carl Zeiss, Thornwood, NY) confocal microscope using objectives of 63X oil (numerical aperture [NA] 1.4). The pinhole was a 1.0 Airy unit. Images were acquired as confocal pictures of 1024×1024 pixels. The excitation light was provided by the 488 nm line of argon lasers for the Alexa-488 fluorophore, the 561 nm line of diode lasers for the Alexa-568 fluorophore, and the 633 nm line of HeNe lasers for the Alexa-633 fluorophore. The images were further improved by reducing blur with deconvolution [24,25]. The Huygens deconvolution software (Scientific Volume Imaging b.v., Netherlands) was used to perform adaptive point spread function deconvolution of the whole confocal picture using 10 iterations. The resulting 32-bit float point two-dimensional image file was imported into Imaris software (64X, 6.1.5, Bitplane, Zurich, Switzerland); from this, a two-dimensional projection picture of the processed image was obtained.

## Results

This study included a total of 82 rats in different age groups. We defined young rats as 3 months of age and old rats as 13 months of age and above.

### The effect of aging on intraocular pressure

**Figure 1:**
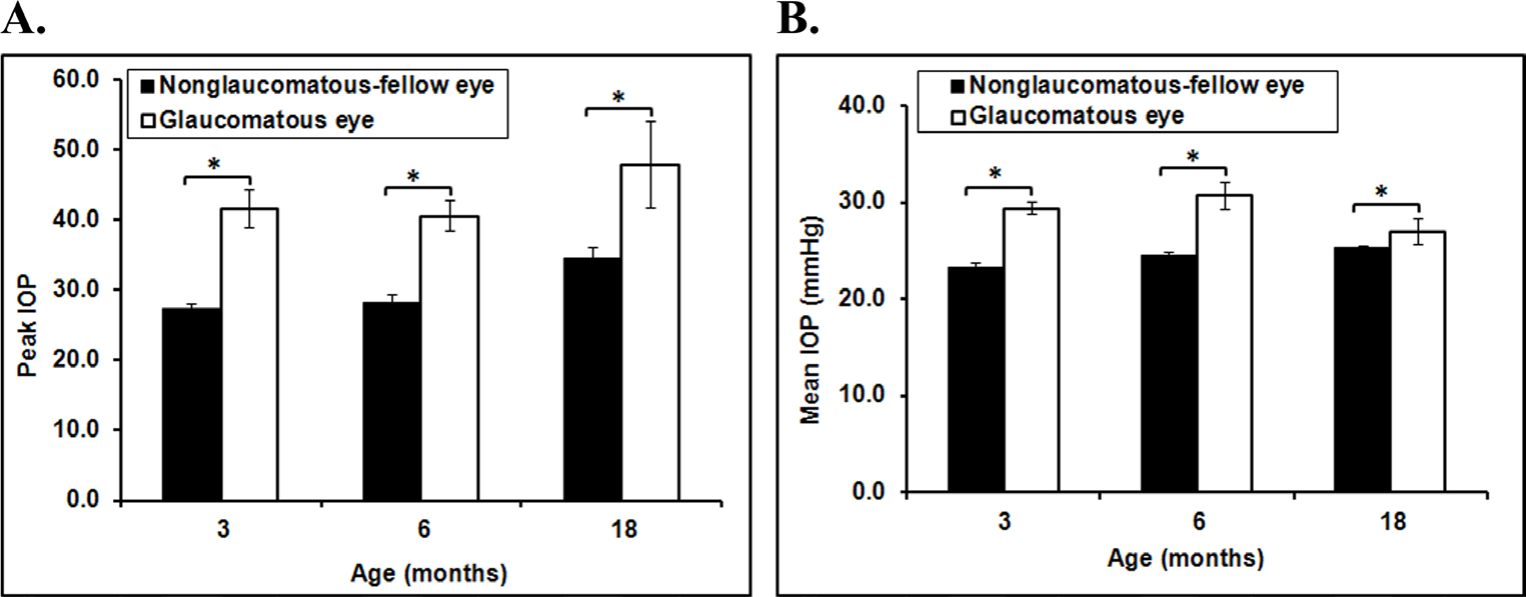
Intraocular pressure in eyes of young and old rats. **A** and **B**: All experimental eyes had significantly elevated intraocular pressure (IOP) compared to their control fellow eyes. There was no significant difference in mean or peak IOP between young and old rats. Data presented as SEM, n=8, *=p<0.05.

All experimental eyes had significantly elevated IOP compared to their control fellow eyes (increase in IOP >10 mmHg; Figure 1A and 1B). The IOP returned to baseline by 2–3 weeks in most animals. The peak IOP was significantly increased in the glaucomatous eyes compared to the control eyes in each age group (n=4–8 rats in each age group, p<0.05, Figure 1A). The mean IOP was also elevated in the glaucomatous eyes of all age groups (n=4–8 in each age group, p<0.01 for the 3 and 6 month olds and p=0.051 for 18 month olds, Figure 1B). There was no significant difference in mean IOP or peak IOP between the age groups.

### The effect of aging on retinal ganglion cell survival

**Figure 2:**
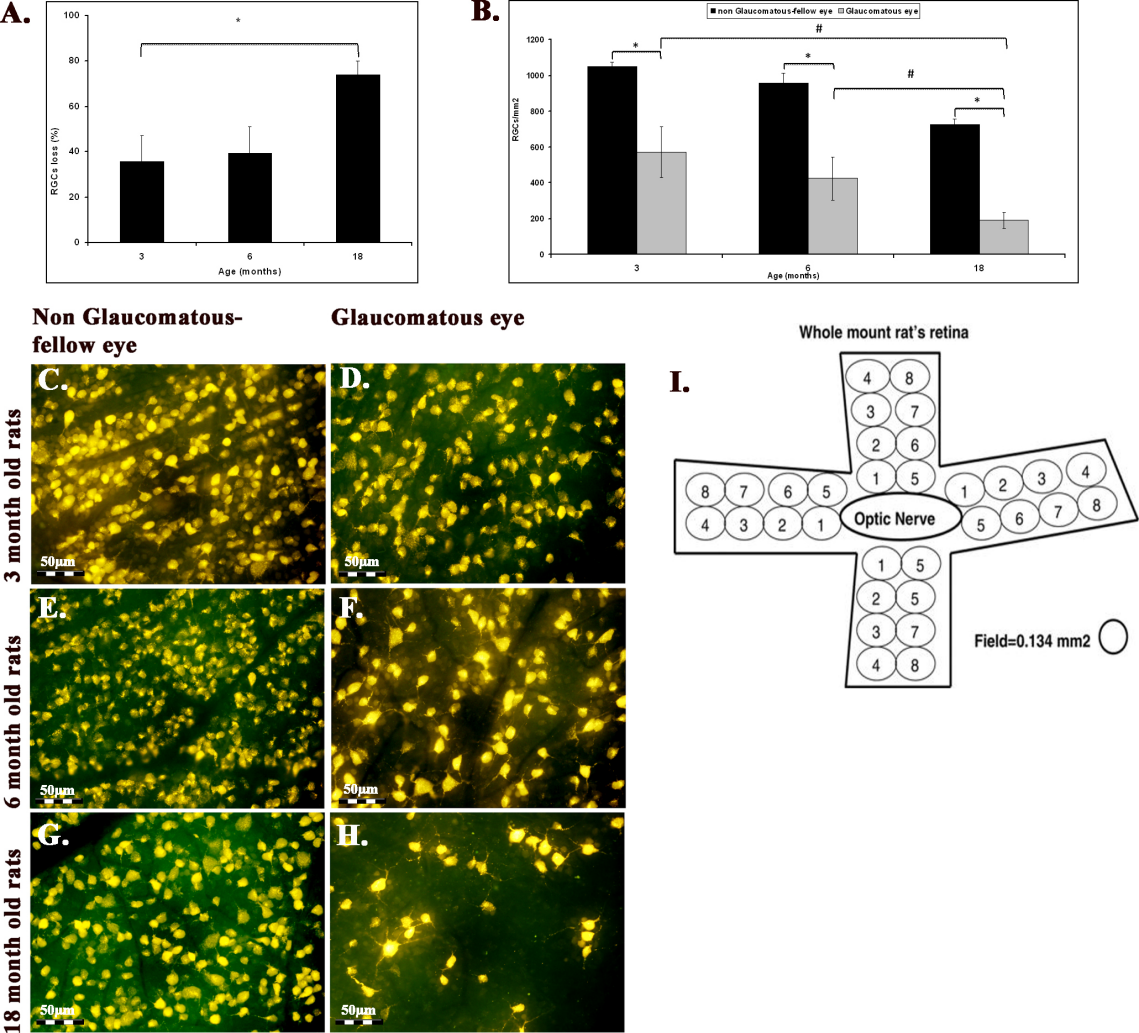
Retinal ganglion cell loss increased with age in both glaucomatous and control fellow eyes. **A**: The mean retinal ganglion cell (RGC) survival 10 weeks after the induction of elevated intraocular pressure (IOP) is shown. There was a significant decrease in RGCs in the control fellow eyes with age (n=4–8 for each age group, data presented as SEM, p=0.002), as well as in the glaucomatous eyes (n=4–8, p=0.048). **B**: The amount of glaucomatous RGC loss increased with age (n=4–8, p=0.05). This progression in RGC loss due to age occurred under similar IOP levels. **C**-**H**: Representative fluorogold images of RGCs 10 weeks after induction of glaucoma in young and old eyes are shown. Magnification 40X. **I**: Labeled RGCs were counted with a 40 super wide field objective along two radii in four directions (i.e., superior, temporal, inferior, and nasal) centered on the position of the optic nerve head.

RGC survival was evaluated at 10 weeks after the induction of elevated IOP. There was a significant decrease in the RGC number with age in the control fellow eyes: It dropped from 1049±26 RGC/mm^2^ at 3 months to 955±57.6 at 6 months and 725±32 RGC/mm^2^ at 18 months (n=4–8 for each age group, p=0.002, analysis of variance [ANOVA], Figure 2A). In addition, elevated IOP induced a significant loss of RGCs in each age group: The number decreased from 669±123 RGC/mm^2^ at 3 months to 486±114 RGC/mm^2^ at 6 months and 189±46.5 RGC/mm^2^ at 18 months (n=4–8, p=0.048, ANOVA; Figure 2A). Thus, there was greater glaucomatous RGC loss with age starting with a 35.8% ± 11.5% loss at 3 months of age to a 39.4% ± 11.7% loss at 6 months and progressing to a 74% ± 6% loss at 18 months (n=4–8, p=0.055, ANOVA, Figure 2B). This age-related progression in RGC loss occurred under similar IOP levels.

### Quantitative polymerase chain reaction array for apoptosis in aged glaucomatous eyes

PCR array results revealed potential gene expression changes that can shed light on the causes for the increased susceptibility of aged RGCs to injury. Genes that were up- or downregulated with at least a twofold change are presented in bold in Table 2. Twenty genes were upregulated in the 3-month-old rats, 8 genes in the 13 month olds, and 12 in the 18 month olds. Downregulation was observed in 16 genes in the 3 month olds, 29 genes in the 13 month olds, and 4 genes in the 18 month olds.

**Table 2:** Summary of fold regulation change following glaucoma induction

**Symbol**	**Description**	**3 month**	**13 month**	**18 month**	**Unigene**	**Refseq**
**Aven**	Apoptosis, caspase activation inhibitor	***5.37***	***-3.19***	−1.31	Rn.145049	NM_001107757
**Bad**	BCL2-associated agonist of cell death	1.98	***2.47***	−1.02	Rn.36696	NM_022698
**Bcl2**	B-cell CLL/lymphoma 2	***3.46***	1.45	1.14	Rn.9996	NM_016993
**Bcl2a1d**	B-cell leukemia/lymphoma 2 related protein A1d	1.75	1.30	***2.17***	Rn.19770	NM_133416
**Bcl2l1**	Bcl2-like 1	***8.35***	***-2.38***	−1.12	Rn.10323	NM_031535
**Bcl2l11**	BCL2-like 11 (apoptosis facilitator)	−1.70	***-2.18***	−1.46	Rn.82709	NM_022612
**Hrk**	Harakiri, BCL2 interacting protein	−1.09	***-2.93***	1.75	Rn.89639	NM_057130
**Bik**	BCL2-interacting killer (apoptosis-inducing)	***2.68***	1.38	1.08	Rn.38487	NM_053704
**Naip2**	NLR family, apoptosis inhibitory protein 2	***2.91***	1.14	***2.48***	Rn.92423	XM_226742
**Birc3**	Baculoviral IAP repeat-containing 3	−1.58	***-3.64***	***-2.08***	Rn.64578	NM_023987
**Card6**	Caspase recruitment domain family, member 6	1.67	***-2.14***	−1.25	Rn.104526	NM_001106413
**Casp1**	Caspase 1	1.61	***-2.90***	***2.03***	Rn.37508	NM_012762
**Casp4**	Caspase 4, apoptosis-related cysteine peptidase	***11.18***	***2.03***	***2.96***	Rn.16195	NM_053736
**Casp12**	Caspase 12	***-2.40***	***2.35***	1.54	Rn.81078	NM_130422
**Casp14**	Caspase 14	***-2.40***	***-3.20***	−1.24	Rn.198773	XM_234878
**Casp6**	Caspase 6	***6.67***	1.78	1.13	Rn.88160	NM_031775
**Casp7**	Caspase 7	***2.11***	***-3.28***	−1.70	Rn.53995	NM_022260
**Casp8**	Caspase 8	1.00	−1.10	1.55	Rn.54474	NM_022277
**Casp8ap2**	Caspase 8 associated protein 2	***-2.04***	***-3.45***	−1.56	Rn.198715	XM_232860
**Cideb**	Cell death-inducing DFFA-like effector b	***-2.40***	−1.71	−1.00	Rn.204016	NM_001108869
**Dapk1**	Death associated protein kinase 1	***3.31***	***-2.52***	−1.46	Rn.23108	NM_001107335
**Dffa**	DNA fragmentation factor, alpha subunit	***2.02***	***-2.17***	−1.92	Rn.6514	NM_053679
**Dffb**	DNA fragmentation factor, beta polypeptide (caspase-activated DNase)	***-2.40***	***-9.22***	***-3.57***	Rn.67077	NM_053362
**Fadd**	Fas (TNFRSF6)-associated via death domain	1.80	***-2.21***	1.13	Rn.16183	NM_152937
**Faim**	Fas apoptotic inhibitory molecule	***3.29***	***-2.65***	1.14	Rn.106419	NM_080895
**Il10**	Interleukin 10	***-2.40***	1.65	***3.63***	Rn.9868	NM_012854
**Lhx4**	LIM homeobox 4	1.60	***-4.09***	−1.12	Rn.48080	NM_001108348
**Lta**	Lymphotoxin alpha (TNF superfamily, member 1)	***-2.40***	−1.64	***4.12***	Rn.160577	NM_080769
**Ltbr**	Lymphotoxin beta receptor (TNFR superfamily, member3)	***3.12***	***8.95***	1.69	Rn.19329	NM_001008315
**Nfkb1**	Nuclear factor of kappa light polypeptide gene enhancer in B-cells 1	***3.31***	***-2.07***	0.73	Rn.2411	XM_342346
**Nol3**	Nucleolar protein 3 (apoptosis repressor with CARD domain)	***2.12***	***2.08***	1.21	Rn.86956	NM_053516
**Polb**	Polymerase (DNA directed), beta	1.41	***-3.24***	−1.23	Rn.9346	NM_017141
**Prdx2**	Peroxiredoxin 2	***2.05***	***2.92***	1.00	Rn.2511	NM_017169
**Prlr**	Prolactin receptor	***-2.40***	1.50	1.06	Rn.9757	NM_012630
**Prok2**	Prokineticin 2	***-2.40***	1.63	***4.20***	Rn.211872	NM_138852
**Pycard**	PYD and CARD domain containing	***2.58***	***-4.96***	1.69	Rn.7817	NM_172322
**Sphk2**	Sphingosine kinase 2	***-2.16***	***-2.98***	1.01	Rn.41053	NM_001012066
**Tnf**	Tumor necrosis factor (TNF superfamily, member 2)	***2.40***	***2.37***	***2.28***	Rn.2275	NM_012675
**Tnfrsf10b**	Tumor necrosis factor receptor superfamily, member 10b	***-2.01***	***-2.83***	−1.22	Rn.105558	NM_001108873
**Tnfrsf11b**	Tumor necrosis factor receptor superfamily, member 11b	***-2.40***	***3.43***	−1.44	Rn.202973	NM_012870
**Tnfrsf1a**	Tumor necrosis factor receptor superfamily, member 1a	***4.08***	−1.59	***2.18***	Rn.11119	NM_013091
**Tnfrsf1b**	Tumor necrosis factor receptor superfamily, member 1b	***3.08***	***-2.11***	−1.01	Rn.83633	NM_130426
**Cd40**	CD40 molecule, TNF receptor superfamily member 5	1.31	1.69	***3.81***	Rn.25180	NM_134360
**Fas**	Fas (TNF receptor superfamily, member 6)	1.20	***2.21***	−1.16	Rn.162521	NM_139194
**Tnfsf10**	Tumor necrosis factor (ligand) superfamily, member 10	1.84	***2.19***	***-2.22***	Rn.83627	NM_145681
**Tp53**	Tumor protein p53	−1.13	−1.29	***-2.44***	Rn.54443	NM_030989
**Tradd**	TNFRSF1A-associated via death domain	1.58	***-2.52***	1.45	Rn.18545	NM_001100480
**LOC687813**	Similar to Tnf receptor-associated factor 1	***-2.40***	***-10.10***	−1.27	Rn.136874	XM_001080233
**Traf3**	Tnf receptor-associated factor 3	1.22	***-2.51***	−1.09	Rn.12033	NM_001108724
**Traf4**	Tnf receptor associated factor 4	***5.03***	***-2.22***	−1.26	Rn.3219	NM_001107017
**Tp53bp2**	Tumor protein p53 binding protein, 2	***2.81***	***-2.61***	1.07	Rn.50333	XM_223012
**Tp63**	Tumor protein p63	***-2.40***	***-2.78***	***3.88***	Rn.42907	NM_019221
**Tp73**	Tumor protein p73	***-2.40***	1.17	***2.21***	Rn.103860	NM_001108696

The upregulated genes in the 3-month-old group included the Bcl-2 family (Bcl2, Bcl2l1), NLR family apoptosis inhibitory protein 2 (Naip2), caspase family (4, 6, and 7), Fas apoptotic inhibitory molecule (Faim), the tumor necrosis factor (TNF) family (Tnfrsf1a, Tnfrsf1b, and Traf4), and Tp53bp2. The downregulated genes were members of the caspase family (8, 14, and Casp8ap2), TNF family (Tnf, Tnfrsf10b, Tnfrsf11b), Tp63, and Tp73. The upregulated genes in the 13-month-old group were proapoptotic genes that included TNF family members (Tnf, Tnfrsf11b, Tnfsf10, and Fas) and caspase family members (4 and 12; Table 2). The downregulated genes were members of the Bcl-2 family, several caspase family members (1, 14, 7, and 8), and tumor protein p53 (p53) family members (Table 2). The upregulated genes in the 18-month-old group also included TNF family members (Tnf, Tnfrsf1a), several caspase family members (1 and 4) and bcl-2. Among the downregulated genes were DNA fragmentation factor, beta subunit (DffB), and p53.

### Validation of reverse transcription polymerase chain reaction

**Figure 3:**
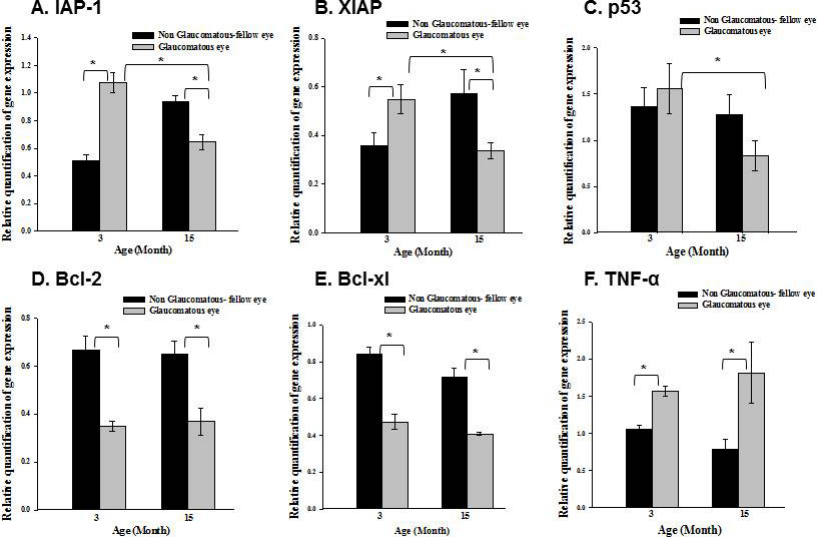
Effect of aging on selected proapoptotic and prosurvival genes. Real-time PCR analysis of selected genes was performed in young (3 months) and old (15 months) rats. The following genes are presented: Inhibitor of apoptosis (IAP)-1 gene (**A**), X-linked IAP (XIAP) gene (**B**); p53 gene (**C**), Bcl-2 gene (**D**), Bcl-xl gene (**E**), tumor necrosis factor (TNF)-α gene (**F**). Interestingly, IAP-1 and XIAP expression was significantly downregulated in the glaucomatous eyes of old rats, but upregulated in young rats. Data represent mean ± standard error of the mean (SEM); n=8; *p<0.05.

The expressions of selected proapoptotic and prosurvival genes were determined using RT–PCR to validate the PCR array results (Figure 3). The most important (and unexpected) finding was the difference between young and old rats in expression levels of the two important prosurvival genes, IAP and XIAP. IAP-1 mRNA levels increased by 111.7±9.5% in the 3-month glaucomatous eyes compared to the fellow control eyes (n=5, p=0.0002), but it decreased by 31.0±8.9% in the 15-month-old rats (n=6, p=0.002; Figure 3A). Another IAP family member, the prosurvival XIAP gene, increased by 53.0±18.2% in the 3-month-old glaucomatous eyes (n=6, p=0.04), but decreased significantly (by 41.6±9.2%) in the 15-month-old eyes (n=7, p=0.04; Figure 3B). There were no changes in P53 mRNA levels in the 3-month-old glaucomatous rats; however, there was a trend toward decline in the 15- month-old eyes (Figure 3C). The P53 mRNA level was reduced by 46.2±19.2% in the 15-month-old eyes (n=5–6, p=0.045) compared to the 3- month-old eyes (Figure 3C). In contrast to the PCR array analysis, Bcl-2 expression was reduced in both the 3- and 15-month-old rats compared to their fellow eye controls (n=5, p=0.00009 and n=7, p=0.0004, respectively, Figure 3D). Bcl-xl mRNA levels were also reduced in both the 3- and 15-month-old rats compared to their fellow eye controls (n=5, p=0.003 and n=7, p=0.007, respectively, Figure 3E). TNF-α mRNA levels increased by 30.5±4.1% in the 3- month-old glaucomatous retinas (n=11, p=0.00003) and by 56.1±6.8% in the 15- month-old glaucomatous retinas (n=6, p=0.04; Figure 3F).

### Immunohistochemical analysis

**Figure 4:**
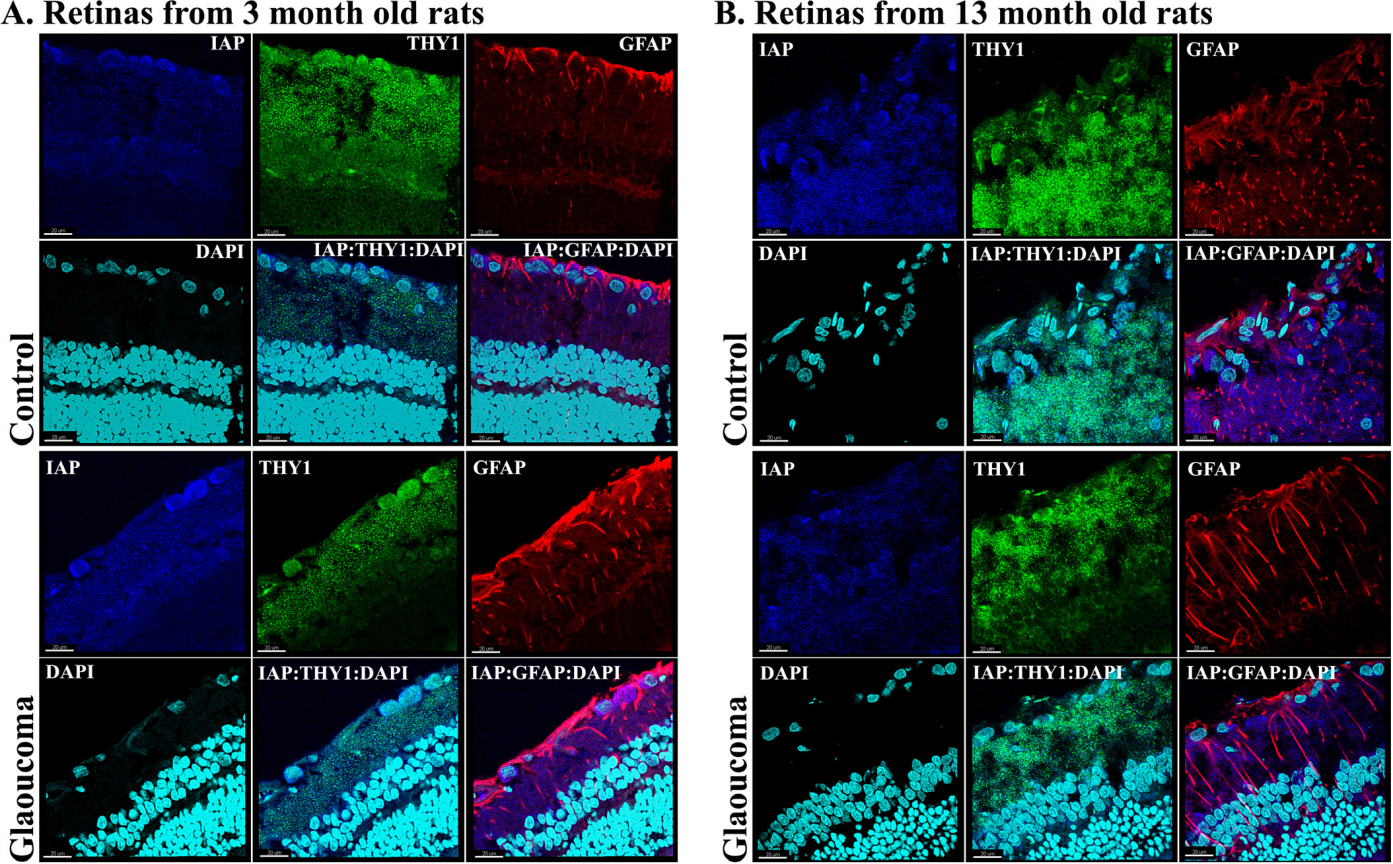
Immunohistochemistry for inhibitor of apoptosis 1, the retinal ganglion cell marker Thy 1, glial fibrillary acidic protein, and 4',6-diamidino-2-phenylindole in retinal cryosections of young and old rats at 8 days after induction of elevated intraocular pressure (IOP). The merged image shows colocalization of IAP with Thy 1 (yellow) and with glial fibrillary acidic protein (GFAP; purple), suggesting that the source for changes in IAP expression is from retinal ganglion cells (RGCs) and glial cells. **A**: In 3-months-old rats, IAP levels increased in glaucomatous eyes as well as staining for GFAP. **B**: IAP-1 staining decreased in old glaucomatous 13-month-old eyes as compared to fellow eyes. Magnification 40X, scale bars: all panels 20 μm.

**Figure 5:**
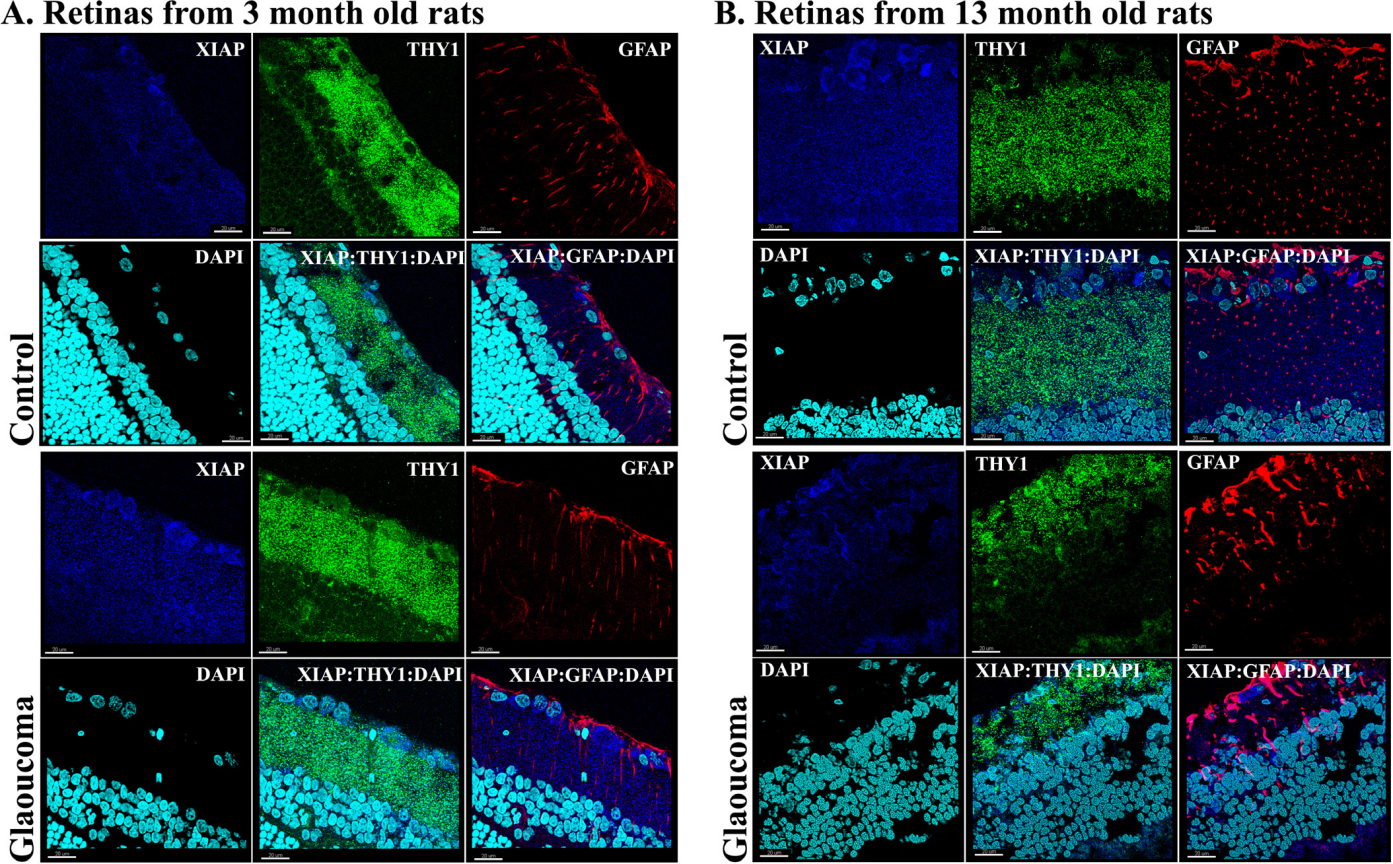
Immunohistochemistry for X-linked inhibitor of apoptosis, Thy 1, glial fibrillary acidic protein, and 4',6-diamidino-2-phenylindole in retinal cryosections of young and old eyes at 8 days after induction of glaucoma. The merged image shows colocalization of X-linked inhibitor of apoptosis (XIAP) with Thy 1 (yellow), suggesting that the source for changes in XIAP expression is in the retinal ganglion cell (RGC) layer. **A**: In 3-month-old eyes, XIAP levels were increased as compared to fellow eyes. **B**: In old glaucomatous 13-month-old eyes, XIAP staining decreased in the RGC layer as compared to fellow eyes. Magnification 40X, scale bars: all panels 20 μm.

Both IAP-1 and XIAP proteins were stained with Thy 1, a marker of RGC cells, and with GFAP, a marker of astrocytes, to investigate and localize any changes that occurred at their protein level. Labeling for IAP-1 was detected in the RGC layer, as well as in other layers of the retina. The intense labeling for IAP in the RGC layer increased in the glaucomatous eyes of 3-month-old rats compared to fellow control eyes and decreased in the 13-month-old rats (Figure 4). Staining for IAP-1, Thy 1, and GFAP suggested that RGCs are the main source for changes in IAP-1 expression. The merged image demonstrated colocalization of IAP-1 with Thy 1 (yellow) and with GFAP (purple). Similarly, staining for XIAP, another member of the IAP family, exhibited an increased in the 3-month-old glaucomatous eyes (Figure 5), but not in the 13-month-old eyes, supporting our RT–PCR data. Staining for XIAP, Thy 1, and GFAP suggested that most of the XIAP secretion came from RGCs (Figure 5). There is clear colocalization of XIAP and Thy 1 (yellow) in the merged image but almost no colocalization of XIAP and GFAP (purple).

## Discussion

The results of this study demonstrated that the rate of RGC damage in glaucomatous eyes increased with age under conditions of similar IOP levels. There was a significant natural loss of RGCs with age in the normal eyes, but this loss increased significantly when glaucoma was induced. This study also contributed novel information on the pathogenesis of glaucoma. We found that the expression of IAP-1, a major prosurvival gene and a potent caspase inhibitor, acts in opposite directions in young and old glaucomatous rat eyes: It increased significantly in the former and decreased significantly in the latter. Another member of the IAP family, XIAP, showed similar age-related behavior in its expression in glaucomatous rat eyes. These data suggest that aging impairs the existing endogenous neuroprotective mechanism that is evoked in response to elevated IOP [26,27].

It is well known that a significant decline in the number of RGCs and their axons occurs with aging [28-30], and thinning of the nerve fiber layer with aging has been recorded by optical coherence tomography [31]. There is limited information about glaucomatous damage with aging, but the current study confirmed that glaucomatous loss of RGCs with age is accelerated beyond the effect of natural RGC loss.

We showed previously that there is simultaneous upregulation of proapoptotic and prosurvival genes, including upregulation of the prosurvival gene IAP-1 and p53 family proapoptotic genes, in glaucoma and optic nerve transection [26]. IAP proteins, as their name implies, confer protection from death-inducing signals by inhibition of diverse apoptosis mediators such as caspase, 3, 6, 7, and 9 [32]. XIAP is the best characterized IAP and the most powerful suppressor of apoptosis [33,34]. In the current study, XIAP and IAP-1 expressions decreased in the glaucomatous retinas of the older eyes, whereas XIAP and IAP-1 expressions increased in the younger eyes, suggesting that inhibition of apoptosis is compromised with age. Members of the IAP family were suggested to play a role in aging [35]. Lymphocytes from elderly humans have been found to express significantly less cellular IAP2 (cIAP2) [35], suggesting that decreased cIAP2 may play a role in increased apoptosis in aged humans. In addition, IAP proteins have been associated with neurodegenerative diseases: NAIP was found to be decreased in Alzheimer disease patients, suggesting that decreased NAIP may place neurons at risk for the development of tangles and apoptosis [36].

IAP family members were found to regulate signaling pathways that activate nuclear factor κB (NFκB), which in turn drives the expression of genes involved in inflammation, immunity, cell migration, and cell survival [37]. IAPs were also identified as ubiquitin-binding proteins contributing to cell survival through NFκB [38]. The connection between NFκB and IAP was further supported by data from studies showing that members of the IAP family of proteins, specifically c-IAP2 and XIAP, are downstream targets of activated NFκB and play a role in antiapoptotic activity [39]. Our PCR array results demonstrated that Nfkb1 levels increased in 3-month-old glaucomatous eyes and decreased in 13-month-old glaucomatous eyes, with no change in 18-month-old glaucomatous eyes. This increase in Nfkb1 observed in the young retinas could have derived from activation of the immune/inflammatory response in glaucoma. It is suggested that this signaling pathway is impaired with age, resulting in a loss of IAP expression and increasing the extent of glaucomatous damage.

Retinal changes in gene expression in glaucoma can originate from various cells types. It is well known that glial and other inflammatory cells are involved in glaucomatous damage. The results of our immunohistochemistry analysis suggest that the changes in IAP-1 and XIAP protein expression were localized to the RGCs and glial cells.

It is now believed that inflammation plays an important role in the development and progression of glaucoma, and several reports have linked TNF-α to glaucoma injury [40-42]. In the current study, the TNF-α expression level increased significantly in the glaucomatous eyes of both the young and old rats, with no effect of aging on TNF-α itself. Our PCR array results yielded no consistent data to suggest any involvement of the TNF family or receptors predisposing RGCs to increased damage with age.

Another interesting signaling pathway that was of particular interest to us was the p53 pathway. We found that p53 gene levels decreased in the glaucomatous eyes of old animals compared to young animals. Studies on the role of p53 in glaucoma suggested that it was involved in the pathogenesis of glaucoma [26,43-45]. We had earlier reported that proapoptotic genes from the p53 pathway, Ei24 and Gadd45a, were upregulated, but that the p53 gene itself stayed unchanged in optic nerve transection and experimental glaucomatous eyes [23]. Thus, the reduced levels of p53 found in this study in the glaucomatous eyes of older rats could be related to the parameter of aging. Indeed, other researchers showed that p53 could act as a potential regulator of organismal aging in mice [46,47]. The low expressions of the DffB and p53 genes in the glaucomatous eyes of the old rats in this study suggest impairment of survival signals in the progression of glaucoma.

Members of the Bcl-2 family are pivotal regulators of the apoptotic process [48], and they play a major role in the apoptosis process of RGCs in glaucoma. However, their expression levels were found to be unaffected by age in glaucoma.

To summarize, this study targeted potential the prosurvival and proapoptotic signaling pathways, which play major roles in glaucomatous damage in young and old rats. Our finding that aging impairs the existing endogenous neuroprotective mechanism of RGCs in glaucoma is novel and opens new directions for further investigations. This enables targeting of specific prosurvival factors or signaling pathways with impaired activity in the retina of old glaucomatous rats to rescue the optic nerve in glaucoma. Further studies on the augmentation of the expression of IAP family members in old glaucomatous rats are underway.
